# Characterization of vascular patterns in endometrial cancer via optical resolution photoacoustic microscopy

**DOI:** 10.1117/1.JBO.31.4.045002

**Published:** 2026-04-29

**Authors:** Lukai Wang, Yixiao Lin, Sanskar Thakur, Jinhua Xu, Quing Zhu

**Affiliations:** a Washington University in St. Louis, Imaging Science Program, St. Louis, Missouri, United States; b Washington University in St. Louis, Department of Biomedical Engineering, St. Louis, Missouri, United States; c Washington University in St. Louis, Mallinckrodt Institute of Radiology, School of Medicine, St. Louis, Missouri, United States

**Keywords:** optical-resolution photoacoustic microscopy, endometrial cancer, microvascular imaging, gynecologic oncology diagnostics, similarity network analysis

## Abstract

**Significance:**

Accurate classification of endometrial pathology is clinically challenging due to the heterogeneous and focal nature of precancerous and malignant lesions. Vascular remodeling is closely linked to tumor progression and may serve as a biomarker for malignancy.

**Aim:**

We aim to characterize a label-free optical-resolution photoacoustic microscopy (OR-PAM) approach for high-resolution imaging and quantitative characterization and separability assessment of endometrial vasculature.

**Approach:**

A custom-built OR-PAM system was used to image 34 fresh uterus samples with histologically confirmed diagnoses: normal, benign, endometrial intraepithelial neoplasia (EIN), and endometrial cancer (EC). Thirty-one quantitative vascular features were extracted from structural and spectral analyses of the photoacoustic data, and five statistically significant and minimally correlated features were selected for the separability assessment framework. A pairwise cosine similarity matrix based on these features was computed to construct a weighted similarity network, which was embedded into a two-dimensional (2D) space with a force-directed layout. A logistic regression boundary was applied to the 2D embedding to evaluate separability between normal/benign and EC/EIN clusters. A logistic regression classifier was developed from a cosine similarity matrix and cross-validated using a leave-one-out strategy.

**Results:**

The cosine-similarity network graph placed 39 of 40 images on the expected side of the separation boundary. The logistic regression classifier yielded an area under the ROC curve (AUC) of 0.943, demonstrating strong discrimination between normal/benign and EC/EIN groups.

**Conclusions:**

OR-PAM combined with imaging feature analysis enables robust differentiation of endometrial pathologies and demonstrates potential as a noninvasive optical biopsy tool for endometrial assessment.

## 1 Introduction

Endometrial cancer (EC) is the most common gynecological cancer in high-income countries, and its incidence is rising globally.^1^ In the United States, an estimated 69,120 new cases and 13,860 deaths are projected for 2025, with annual incidence increasing by 1% to 3%.^2^ Clinical outcomes vary significantly depending on the stage at diagnosis: about two-thirds of patients are diagnosed at an early stage and have favorable prognoses, with 5-year overall survival (OS) rates around 81%, whereas OS drops significantly to 17% and 15% for those diagnosed at stage IVA or IVB, respectively.^3^ Endometrial hyperplasia, a common precancerous condition, exhibits varying malignant potential based on histology. Fewer than 5% of hyperplasia cases without atypia progress to cancer over 20 years,^4^ whereas ∼23% to 28% of patients with atypical hyperplasia, classified as Endometrial Intraepithelial Neoplasia (EIN), develop carcinoma over a similar timeframe.^5^ These statistics highlight the need for accurate and timely detection of endometrial lesions to guide appropriate clinical intervention.

Despite high sensitivity, current diagnostic pathways for endometrial cancer rely on invasive tissue sampling, often triggered by nonspecific findings on transvaginal ultrasound.^6^ These procedures can be painful, prone to sampling errors, and limited by poor specificity, especially in premenopausal women. Patients report a wide range of experiences with intrauterine procedures, from mild discomfort to intense pain, and in some cases, general anesthesia is needed to complete the examination. As such, there is a pressing need for improved diagnostic approaches that provide more accurate risk stratification and reduce unnecessary invasive procedures.

A promising direction in diagnostic innovation is the development of high-resolution imaging techniques that provide quantitative, pathology-relevant information at the point of care. Photoacoustic imaging is a hybrid modality that combines optical absorption contrast with ultrasonic detection to provide structural and functional information. It includes photoacoustic tomography (PAT), which achieves centimeter-scale imaging depth with ultrasound resolution, and photoacoustic microscopy (PAM), which offers higher spatial resolution through local light excitation and high-frequency ultrasound detection. In PAM, optical excitation and ultrasonic detection are typically coaxial and confocal. Two configurations are commonly used: acoustic-resolution PAM (AR-PAM) relies on tight acoustic focusing, enabling deeper tissue imaging (typically in several millimeters in biological tissues), whereas optical-resolution PAM (OR-PAM) applies optical focusing to achieve micron-scale lateral resolution but with limited imaging depth (typically <1  mm in biological tissue) due to optical scattering and absorption.^7^ PAT is commonly used for deeper tissue imaging at the organ level, such as breast,^8^^,^^9^ ovary,^10^^,^^11^ and thyroid,^12^ whereas PAM is preferred when high resolution of subsurface vasculature imaging is needed.^13^^,^^14^

Because microvascular remodeling in epithelial tissues occurs at the capillary scale, imaging modalities capable of resolving individual microvessels are particularly valuable for disease characterization. OR-PAM uniquely fulfills this requirement by enabling vascular imaging with micrometer resolution. Previous studies have demonstrated the versatility of OR-PAM in capturing capillary-level vascular features with high resolution and diagnostic relevance.^14^^–^^16^ Specifically, OR-PAM has been shown to distinguish between benign and malignant ovarian lesions based on quantifiable vascular features, as demonstrated in prior studies from our group.^17^^–^^19^ Beyond that, OR-PAM has also been explored by other groups for clinical applications such as prostate cancer monitoring,^20^ early oral cancer detection,^21^ cutaneous microvasculature,^22^ and breast tissue cancer margin^23^ imaging. Importantly, the endometrium presents a biologically and structurally distinct vascular environment compared with the ovary, characterized by cyclical hormonal remodeling, stromal organization, and premalignant progression pathways that have not previously been investigated using OR-PAM. The endometrium consists of a superficial functional layer supplied by spiral arteries that branch into a subepithelial capillary plexus near the luminal surface, positioning pathology-relevant microvasculature within the submillimeter penetration depth of OR-PAM.^24^ By capturing diagnostically relevant vascular patterns without the need for contrast agents or invasive sampling, OR-PAM may serve as a valuable complement to conventional histological methods in the evaluation of endometrial pathology.

In this study, we used a custom-built OR-PAM system to image the endometrial cavity of freshly resected uterus samples following hysterectomy, with the goal of differentiating normal/benign endometrium from endometrium with malignant or premalignant pathology (EC and EIN) based on quantitative structural and spectral features. A custom analysis pipeline was developed to extract five quantitative features: the mean frequency of the photoacoustic (PA) signal, which reflects tissue composition^25^^,^^26^; the mean and standard deviation (SD) of vessel diameter, capturing vessel size and heterogeneity; the normalized isolated branch length and the branching interval, both describing vascular morphology. By analyzing these features across different pathological conditions, this study evaluates the potential of OR-PAM as a label-free optical biopsy tool for accurate endometrial lesion diagnosis and identifies interpretable vascular biomarkers associated with premalignant progression or malignancy in the endometrium to enable biologically grounded risk stratification, with future potential for integration into catheter-based imaging platforms for minimally invasive assessment. To the best of our knowledge, this is the first study to utilize OR-PAM for quantitative vascular profiling of endometrial cancer and its premalignant precursors.

## 2 Methods

### 2.1 Imaging System

A previously described custom-built OR-PAM system^17^ was used to image the endometrium of uterus samples immediately after hysterectomy. The system employs a 532-nm pulsed laser for optical excitation and a customized high-frequency piezoelectric ultrasonic transducer (center frequency 40 MHz, −6  dB bandwidth of 75%, and focal depth 6.35 mm) to detect the generated PA signals. A motorized two-dimensional (2D) scanning stage raster-scans the focused beam across the region of interest (ROI) with a step size of 3  μm, enabling capillary-scale visualization based on hemoglobin’s intrinsic absorption. The system achieves a resolution of 3  μm. Each B-scan comprised 1000 A-lines, and volumetric C-scans consisted of 1000 to 5000 B-scans, depending on the ROI size.

### 2.2 Patient Recruitment

This study was approved by the Institutional Review Board of Washington University School of Medicine. Between February 2025 and June 2025, 50 patients provided informed consent and were enrolled in the study. Sixteen patients were excluded from the final analysis due to excessive surface bleeding. From the remaining 34 patients, imaging was performed on the anterior and/or posterior endometrium depending on imaging time constraints. In cases where the two sides exhibited different pathologies, each was treated as an independent data point, resulting in 40 analyzable C-scan images: 10 from normal tissue, 25 from EC, and 5 from EIN, as shown in Table 1.

**Table 1:** Summary of sample characteristics and pathological diagnosis after surgery.

Sample groups (n=40^a^)	Mean age: 60; Range: 35 to 86	Histological diagnosis based on hematoxylin and eosin
EC (n=25)	63 (35 to 86)	FIGO grade 1 (n=21)
		FIGO grade 2 (n=4)
EIN (n=5)	60 (47 to 83)	EIN (n=2)
		EIN with treatment effects (n=3)
Benign (n=3)	61 (53 to 67)	Polyps and cystic atrophy (n=3)
Normal (n=7)	54 (41 to 81)	Inactive endometrium (n=4)
		Proliferative endometrium (n=3)

a n is the number of C-scans

### 2.3 Imaging Procedure

Freshly resected uterus samples were imaged *ex vivo* immediately after hysterectomy. The samples were bivalved to expose the endometrial surface for imaging. ROIs on the anterior or posterior sides were visually identified. A real-time LabVIEW graphical user interface was used to control the scan parameters and provide real-time image display.

### 2.4 Data Processing

**Fig. 1:**
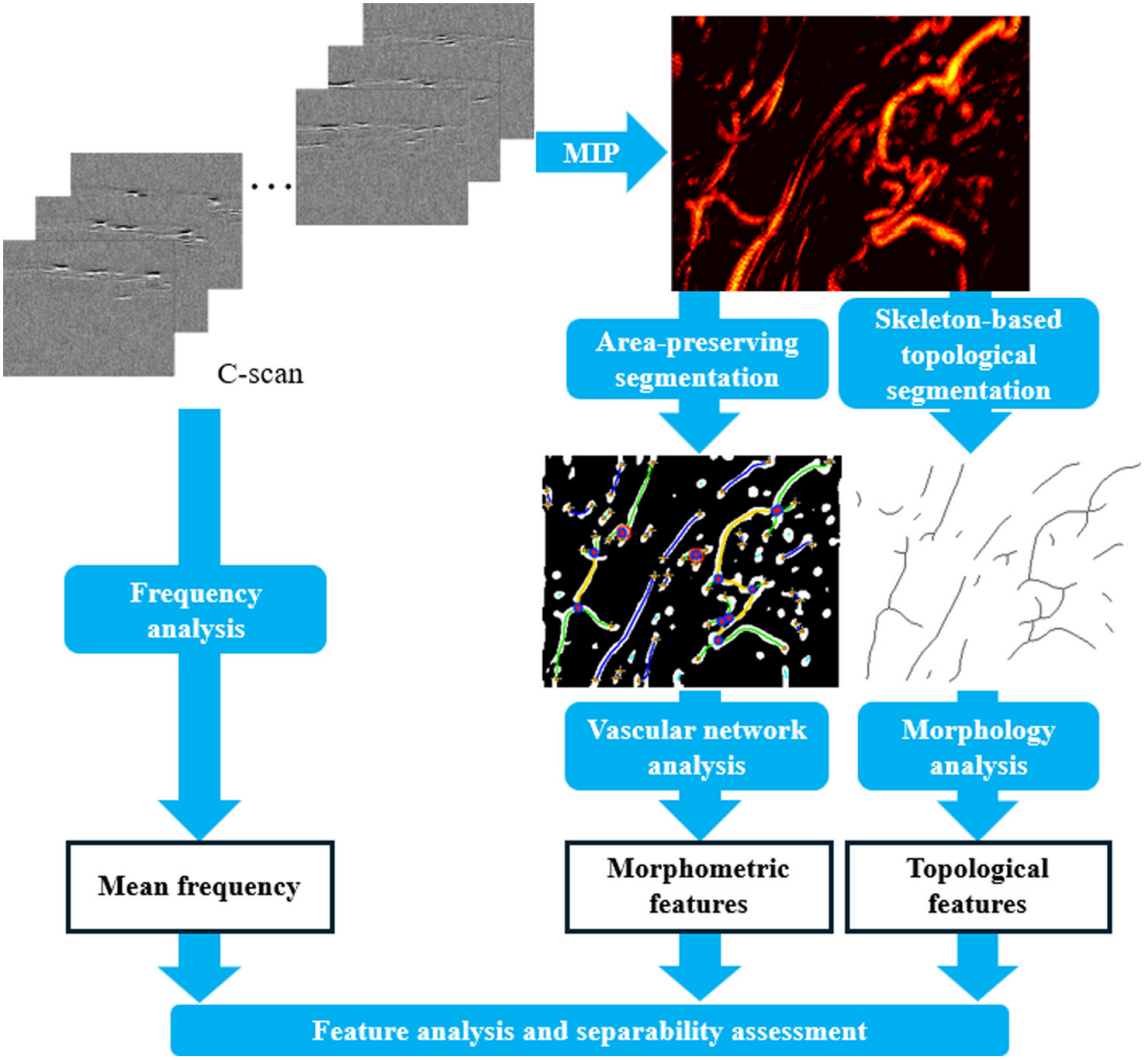
OR-PAM feature extraction pipeline. OR-PAM C-scan datasets are processed through two parallel branches: (1) MIP images are generated for structural analysis. These MIPs undergo two complementary segmentation strategies: area-preserving segmentation, which retains vessel cross-sectional information for morphology analysis to quantify morphometric features such as vessel diameter and shape metrics, and skeleton-based topological segmentation, which reduces vessels to centerlines for vascular network analysis to quantify topological features such as branching, connectivity, and network topology; (2) frequency analysis is performed directly on the RF C-scan data to calculate the mean frequency. Features from all branches are combined for downstream statistical analysis and separability assessment. (a) Normal endometrium. (b) Grade 1 endometrioid adenocarcinoma. (c) Atypical hyperplasia (EIN).

Raw OR-PAM volumetric datasets were processed with a custom pipeline to extract structural and spectral features as shown in Fig. 1. The radiofrequency (RF) signals were bandpass filtered (30 kHz to 50 MHz), and Hilbert transformed to compute the PA envelope amplitude. A 45-dB display dynamic range was applied, and maximum intensity projections (MIP) were generated for visualization. To characterize vascular structure quantitatively, two parallel analysis pipelines were employed.

#### 2.4.1 Morphology analysis

Vascular morphology in each MIP was quantified with the DiameterJ Fiji plugin in ImageJ.^27^ Images were first preprocessed to enhance local vessel contrast and suppress background variations using contrast enhancement and background subtraction. The processed images were then segmented into binary vessel masks using DiameterJ’s segmentation algorithm. DiameterJ then estimates vessel diameters using a distance-transform–based approach^28^: the binary vasculature is skeletonized to extract vessel centerlines, and the local radius at each centerline pixel is computed as the shortest Euclidean distance to the vessel boundary. Vessel diameter is defined as twice this radius, producing a spatially resolved diameter map for each vessel segment. Spatial calibration was applied using known pixel-to-micron conversion factors to report diameters in physical units. From the resulting diameter distributions, 11 morphometric features, including mean vessel diameter and standard deviation, were computed for each image.

#### 2.4.2 Vascular Network Analysis

The same ROI images were processed with the Angiogenesis Analyzer plugin in Fiji to evaluate vascular network topology. Images were first binarized and skeletonized with an iterative thinning procedure. The plugin automatically identifies vascular elements including endpoints, branching nodes, segments, master segments, and isolated segments based on pixel connectivity and network topology rules. Quantitative features such as the number of branches, total branch length, and branching interval were computed from the extracted skeleton. Features related to vessel counts or cumulative vessel length were normalized by ROI area to ensure comparability across samples.

In parallel, the RF signals were analyzed in the frequency domain: a pixel-wise power spectral map was computed for each image using robust multipixel sliding averaging.^29^ The resulting spectral map was averaged across the entire image to obtain the mean frequency of the PA signals for the corresponding endometrium sample by calculating the weighted mean of the power spectrum. This mean frequency reflects the effective absorber dimensions and tissue microstructure, whereas the measured spectrum is also shaped by the bandwidth of the transducer, which was kept constant across all samples.

### 2.5 Feature Selection and Classification

Thirty-one quantitative features were extracted from frequency analysis, morphology analysis, and vascular network analysis. A complete list of all extracted features and their descriptions is provided in the Supplementary Material. Features discriminating between EC/EIN and normal/benign cases were first screened using Welch’s t-test. Redundancy was subsequently reduced by pairwise correlation analysis. The five most statistically significant and mutually uncorrelated features were retained for the separation framework.

To visualize interimage relationships and assess separability, a cosine similarity matrix was computed using the five selected features, and a network graph was constructed. Each feature vector was L2-normalized, and pairwise cosine similarities were computed as the inner product between normalized feature vectors from each image. In the resulting similarity network, each node represents a normalized feature vector and edges reflect cosine similarity between them. To obtain a low-dimensional representation, the similarity graph was embedded onto a 2D plane using a force–directed layout algorithm.^30^ In this approach, nodes connected by higher similarity weights exert stronger attractive forces, while all nodes experience repulsive forces. Node positions are iteratively updated to minimize the system energy, producing a 2D embedding that preserves relational similarity structure rather than performing linear dimensionality reduction such as principal component analysis. To visualize group separability within the embedding space, a binary logistic regression boundary was applied to the resulting 2D coordinates.

To evaluate classification performance, a logistic regression classifier was implemented using a leave-one-out cross-validation (LOOCV) strategy on the 40×40 cosine-similarity matrix derived from feature vectors. In each iteration, one feature vector was held out for testing and four summary attributes were computed from the remaining 39×39 similarity matrix. For each training feature vector, the mean and standard deviation of similarity to the normal/benign cluster and to the EC/EIN cluster were calculated, forming a 39×4 training matrix. The logistic-regression model was trained 40 times on these matrices, and the held-out feature vector was tested in each iteration to estimate overall performance.

This framework integrates unsupervised relational visualization with supervised linear separation and internal cross-validation to characterize vascular feature discriminability between normal/benign and EC/EIN samples.

## 3 Results

**Fig. 2:**
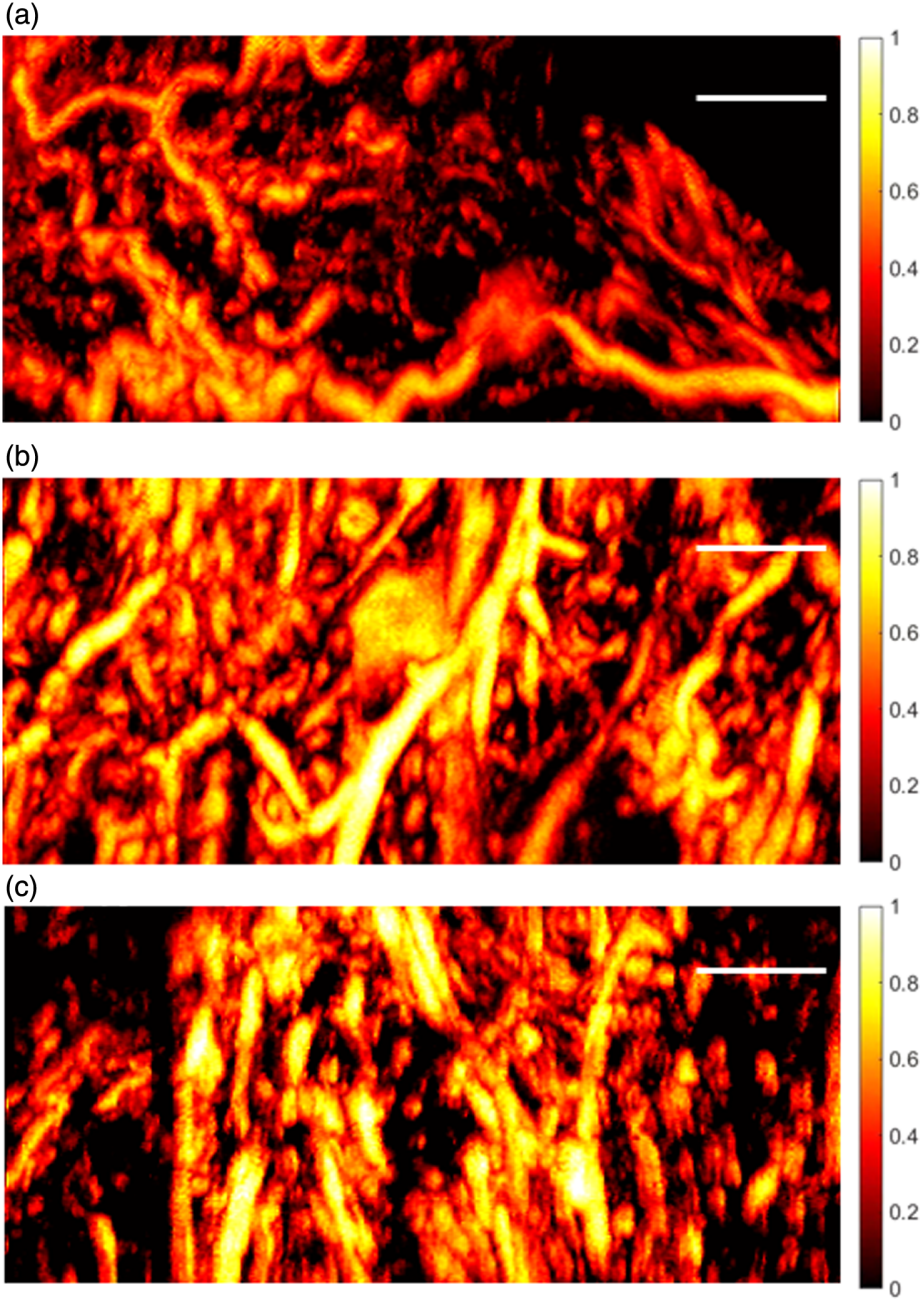
Representative MIP images of normal endometrium (a), EC (b), and EIN (c). White solid bars indicate a scale of 1 mm.

Following image acquisition and feature extraction, we assessed structural and spectral differences across pathology groups and evaluated the discriminative capability of the selected features. Representative MIP images in Fig. 2 show that, compared to the sparser and more well-organized vasculature in normal endometrium, EC and EIN exhibit dense, disorganized, and enlarged vessels, consistent with tumor-associated vascular abnormalities.

**Fig. 3:**
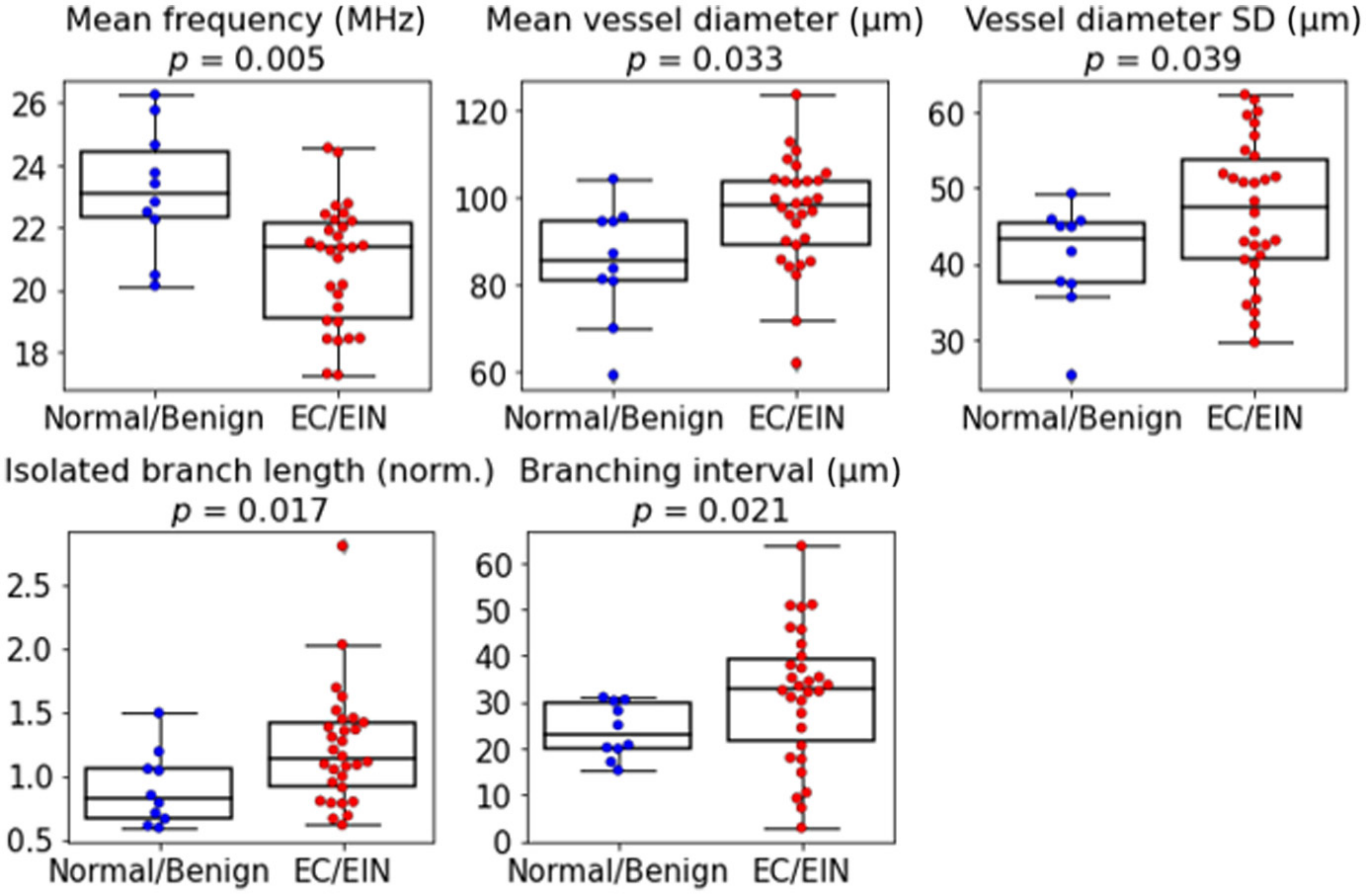
Boxplots of the five selected features. P-values from Welch’s t tests are shown above each subplot.

Based on statistical significance and low inter–feature redundancy, five features were selected for the separation framework: mean frequency, mean and standard deviation (SD) of vessel diameter, normalized isolated branch length, and branching interval. In this context, a branch refers to a continuous vessel segment between two bifurcation points or between a bifurcation and a terminal end. The branching interval represents the average centerline distance between adjacent branching nodes along the vascular network skeleton. As shown in Fig. 3, all five features demonstrate significant differences between normal/benign and EC/EIN groups from Welch’s t-test. The pairwise correlation matrix of the selected features, provided in the Supplementary Material, reveals only low to moderate correlations, indicating minimal redundancy among the selected features. Collectively, these results highlight the complementary nature of structural and spectral features captured by OR-PAM in differentiating malignant and premalignant endometrial tissue from normal and benign endometrium.

**Fig. 4:**
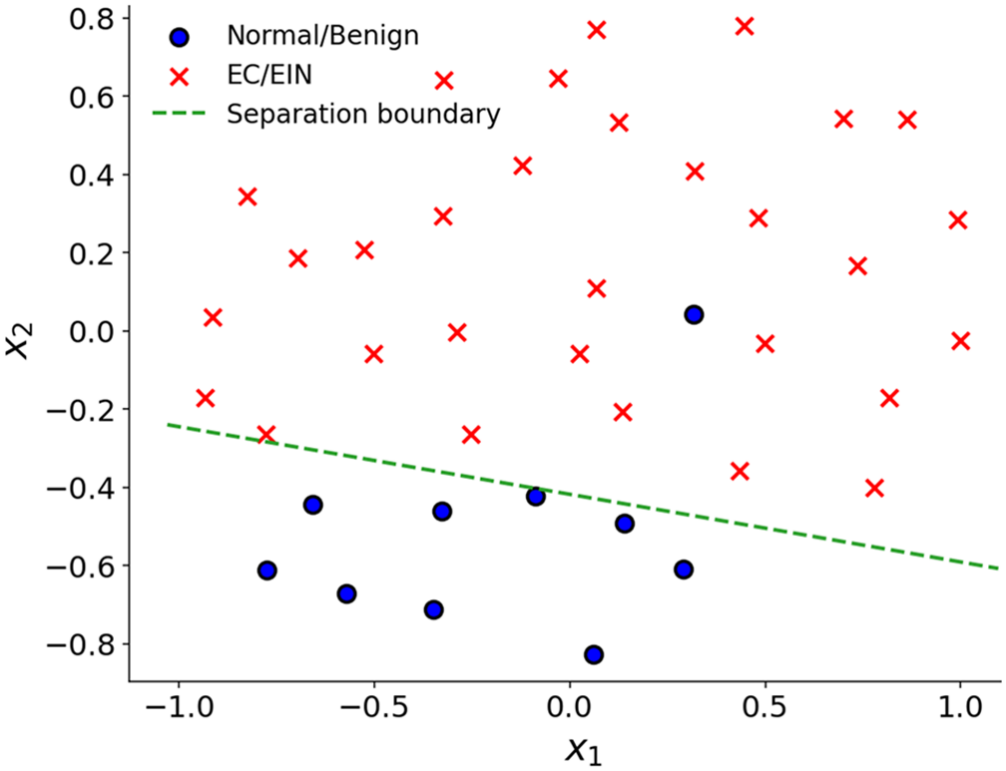
2D embedding of vascular features based on cosine similarity. Each node represents one feature vector projected onto a 2D plane using a force-directed layout. The dashed green line represents the linear separation boundary obtained from logistic regression, illustrating the discriminative capability of the selected five-feature set. All 30 EC/EIN images lie on one side of the boundary, whereas 9 of 10 normal/benign images lie on the other side.

The separation framework based on the five selected features demonstrated clear separation between EC/EIN and normal/benign cases, as shown in Fig. 4. All 30 EC/EIN images were positioned on one side of the separation boundary, whereas 9 out of 10 normal/benign images were positioned on the other side. These results indicate strong feature-level separability within the embedding space and suggest that the selected vascular features capture biologically meaningful trends associated with premalignant and malignant progression.

**Fig. 5:**
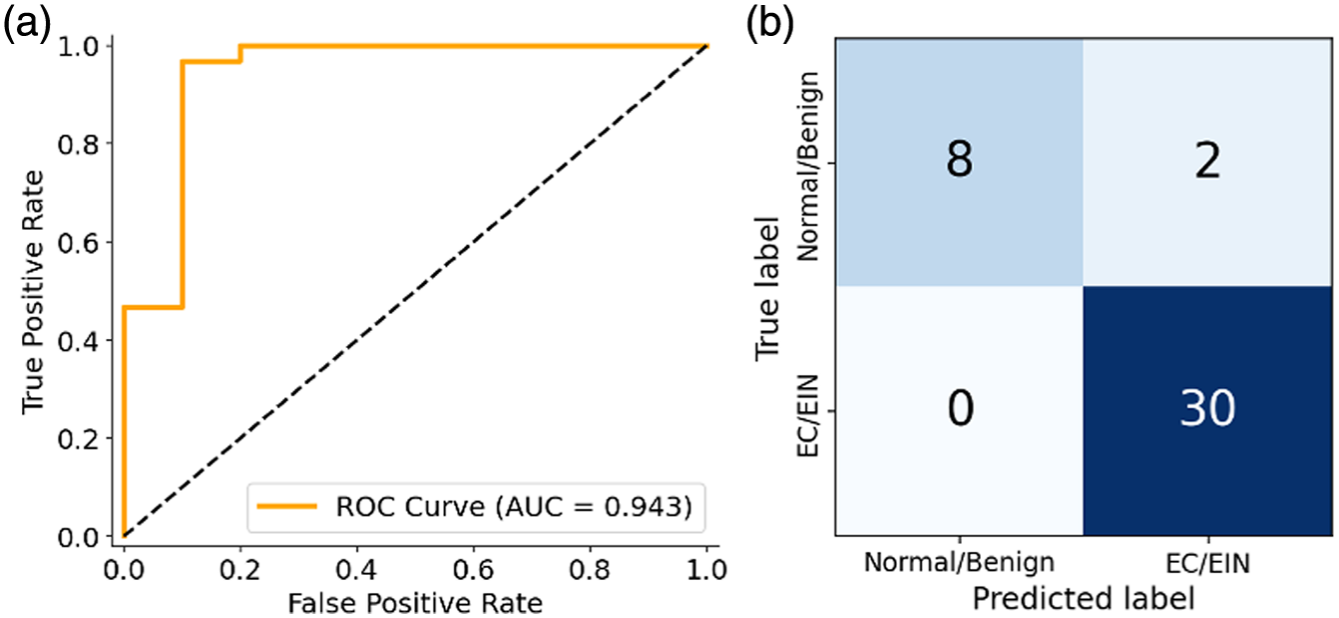
LOOCV performance of the logistic regression classifier. (a) Receiver operating characteristic (ROC) curve generated from LOOCV predictions, yielding an area under the curve (AUC) of 0.943. (b) Confusion matrix summarizing aggregated out-of-sample predictions across all LOOCV iterations, demonstrating 100% sensitivity and 80% specificity.

The logistic regression classifier yielded an AUC of 0.943 [Fig. 5(a)], indicating strong discrimination between EC/EIN and normal/benign cases. Using 0.5 as a threshold for the classifier, we computed the confusion matrix [Fig. 5(b)], which showed 100% sensitivity (30/30 EC/EIN correctly identified) and 80% specificity (8/10 normal/benign correctly identified), corresponding to an overall accuracy of 95%. These findings suggest that the observed separation reflects stable structure within the vascular feature space.

To better understand classification performance, we examined the two normal/benign cases that were misclassified as EC/EIN in the LOOCV analysis. These corresponded to a 66-year-old and a 52-year-old postmenopausal patient. For several vascular features, their values overlapped with the EC/EIN distribution, shifting their combined feature profiles toward the EC/EIN region of the feature space. Boxplots of the five selected features with these cases highlighted are provided in the Supplementary Material.

This observation highlights the partial overlap between vascular patterns in normal/benign and premalignant/malignant endometrium, likely reflecting underlying biological heterogeneity. In the present study, the OR-PAM system operates at a single wavelength, precluding measurement of oxygen saturation, a metabolic biomarker that may provide complementary diagnostic information. Moreover, oxygen saturation measurements are inherently limited in the *ex vivo* setting. Together, these observations motivate future work incorporating multimodal vascular and metabolic features to achieve more robust and generalizable diagnostic discrimination in patient studies.

## 4 Discussion

This study demonstrates the potential of OR-PAM for quantitative characterization and discrimination of endometrial pathology. A custom image analysis pipeline was developed to extract vascular features, including vessel morphology, network topology, and PA spectral properties, from OR-PAM scans. Using five statistically informative and mutually uncorrelated features, the separation framework achieved near-complete separation on the full dataset (39/40 images correctly positioned). The logistic regression classifier cross-validated with the leave-one-out strategy maintained strong discrimination with an AUC of 0.943, 100% sensitivity, 80% specificity, and 95% overall accuracy.

The selected features reflect biologically meaningful differences in vascular architecture and tissue composition between normal and diseased endometrium. EC and EIN exhibited lower PA frequencies, consistent with enlarged vascular structures and reduced stromal scattering,^25^ along with larger and more heterogeneous vessel diameters. Increased normalized isolated branch length and longer branching intervals indicate a fragmented and less interconnected vascular network, consistent with tumor-associated angiogenesis and stromal remodeling. Normalized isolated branch length can be interpreted as a surrogate of vascular network fragmentation, as higher values reflect a greater proportion of vessel length disconnected from the main network within a fixed area. This interpretation is consistent with Fig. 2, where EC/EIN samples show numerous short, isolated vessel segments, aligning with the expected disorganized angiogenesis in premalignant and malignant endometrium.

In addition, 16 cases (32% of those enrolled) were excluded due to excessive surface bleeding, which hindered visualization of subsurface vasculature. Among these excluded cases, five were premenopausal, suggesting that hormonal status and associated endometrial vascular dynamics may contribute to increased surface bleeding and reduced imaging quality. The current study did not record perimenopausal status and timing of the menstrual cycles for premenopausal women. As hormonal status is known to influence endometrial thickness, vascular density, and bleeding propensity, these factors may contribute to variability in image quality and extracted vascular features, representing a potential confounding factor in the current analysis. Although a quantitative image quality assessment pipeline was implemented to support objective exclusion, this limitation highlights the need for improved imaging protocols or strategies to reduce the effect of surface artifacts and enable more reliable visualization of the underlying vasculature. Details of the image quality control assessment for the current study are provided in the Supplementary Material.

In addition to image quality considerations, the accuracy of vessel segmentation and subsequent skeletonization represents another source of variability in the current analysis. In OR-PAM images, strong signals from surface blood residues, bleeding artifacts, or other high-intensity structures may be misidentified as vascular features by automated segmentation algorithms. As a result, nonvascular signals may be partially included in the segmented masks, leading to imperfect estimation of vessel morphology and network topology. Although widely used Fiji-based plugins were employed for consistency and reproducibility, these approaches are not specifically optimized for OR-PAM data and may be sensitive to such artifacts. Nevertheless, because the same preprocessing and segmentation pipeline was uniformly applied across all samples, the comparative analysis between normal/benign and EC/EIN groups remains internally consistent. The strong separability observed suggests that the extracted features are robust to these segmentation imperfections.

Further, this study did not incorporate depth information due to tissue deformation during imaging and the inherently shallow penetration depth of OR-PAM. Although depth-dependent vascular heterogeneity may carry diagnostic value, robust recovery of this information would require improved normalization or reconstruction strategies to restore microvasculature in its native 3D spatial context.

Future work will account for menopausal status and menstrual cycle timing as control variables, enabling more precise separation of disease-related vascular alterations from physiological hormonal effects.

Clinical translation will require effort on both instrumentation and computational methods. On the instrumentation side, future systems may incorporate co-registered ultrasound with OR-PAM, where ultrasound provides complementary, deeper structural information while OR-PAM offers high-resolution subsurface microvascular characterization. In addition, strategies such as reversible optical clearing^31^ and advanced beam engineering^32^ may help extend the effective imaging depth while maintaining resolution. Further developments, including multiwavelength excitation for functional measurements and miniaturized fiber-based probes compatible with hysteroscopic or intravaginal access, could position the imaging interface directly at the endometrial surface to enable real-time *in vivo* assessment. On the computational side, deep-learning-based analysis strategies trained on spatially limited OR-PAM sampling regions may reduce the required scan area and acquisition time, thereby improving clinical workflow feasibility.^19^ Together, these advances could enable practical deployment of OR-PAM as a minimally invasive optical biopsy tool for endometrial disease diagnostics.

## 5 Conclusion

This study establishes OR-PAM as a promising modality for the quantitative assessment of endometrial pathology, enabling robust differentiation between benign and malignant tissue based on interpretable vascular features. A five-feature separation framework demonstrated strong discrimination between normal/benign and EC/EIN samples, highlighting the clinical relevance of microvascular alterations captured by OR-PAM. To our knowledge, this represents the first application of OR-PAM for quantitative endometrial cancer assessment. These findings support the potential of OR-PAM as a minimally invasive optical biopsy approach for endometrial cancer imaging and diagnosis. Further technical advances, including surface artifact mitigation via machine learning, instrumentation advances, and expansion to larger patient cohorts, will be key to advancing its translational impact.

## Supplementary Material

10.1117/1.JBO.31.4.045002.s01

## Data Availability

The datasets generated and/or analyzed during the current study are not publicly available due to patient privacy concerns, but are available from the corresponding author on reasonable request. Code for data analysis can be found at https://github.com/OpticalUltrasoundImaging/OR-PAM-Endometrial-Cancer.
