# Supplementary material for: Characterization of vascular patterns in endometrial cancer via optical resolution photoacoustic microscopy
Source: J Biomed Opt. 2026 Apr 29;31(4):045002. doi: 10.1117/1.JBO.31.4.045002 (PMC13126656; doi:10.1117/1.JBO.31.4.045002)
Supplement: Supplementary file 1 [file JBO_031_045002_SD001.docx]

**Supplementary Note 1: Surface Bleeding and Image Quality Assessment**

A key challenge in this study is the presence of surface bleeding in some samples. In affected cases, pooled blood at the tissue surface generated strong superficial PA signals that obscured underlying vasculature and compromised feature extraction. To ensure analytic validity, we excluded images with obvious bleeding artifacts based on predefined visual criteria including amorphous high-intensity patches, loss of vessel-like connectivity, and global structural disorganization. To validate that this exclusion process reflected objective differences rather than subjective bias and to enable future automation, we developed an image quality assessment pipeline to quantitatively compare analyzable and non-analyzable images.

The pipeline is summarized in Figure S1. The key difference between analyzable and non-analyzable images is preservation of microvascular architecture: analyzable images show coherent, elongated vessel-like patterns, whereas non-analyzable images appear amorphous and lack network connectivity. OR-PAM images were first contrast-enhanced and filtered with the Tubeness filter to highlight linear vessel-like patterns. The resulting images were then binarized, and quantitative morphological features of segmented regions were extracted using Fiji’s Analyze Particles tool. This tool detects each segmented object, outlines it (yellow in the final panel of Figure S1) and calculates shape and size features such as area, perimeter, Feret diameter and circularity. Typically, analyzable images contain fewer, larger, tubular objects, whereas non-analyzable images contain numerous small, irregular fragments with poor connectivity. These differences in segmentation patterns yield distinct distributions across particle count, area, elongation, and other features. The extracted morphological features therefore encode key structural distinctions between analyzable and non-analyzable images and were used as input features for downstream classification of image quality.


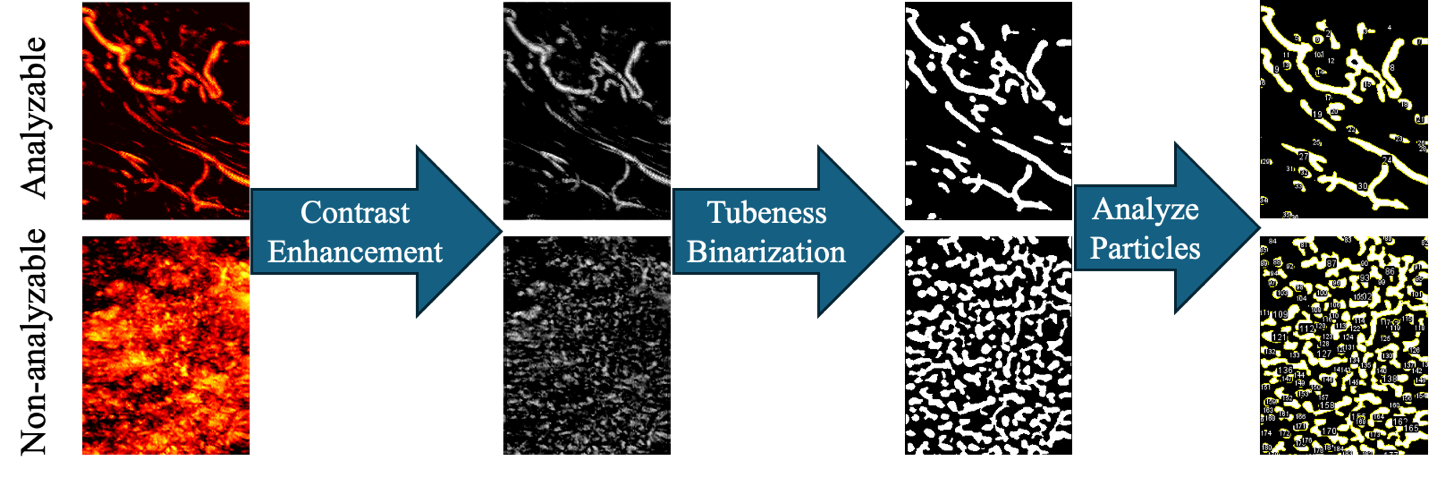


Figure S1. Image quality analysis pipeline. Representative examples of analyzable (top row) and non-analyzable (bottom row) OR-PAM images are shown across each processing stage. After contrast enhancement, images were filtered using the Tubeness filter to highlight vessel-like structures, then binarized to generate binary masks. These masks were analyzed using Fiji’s Analyze Particles tool to extract quantitative morphological features such as area, perimeter, Feret diameter, and shape features. These features served as the input for downstream classification of image quality.

Among the morphological features computed with the Analyze Particles tool, four representative and interpretable features were selected for detailed comparison as shown in Figure S2: segmentation density, segmented area density, Feret diameter, and average particle size. Segmentation density reflects the number of segmented areas per unit area, while segmented area density quantifies the total area covered by these objects—both indicating the degree of structural clutter. Feret diameter measures the longest axis of a segmented region, approximating its elongation, and average particle size captures the mean area of individual structures. Non-analyzable images showed higher segmentation and area densities, consistent with numerous small, amorphous regions from surface bleeding. In contrast, analyzable images exhibited larger Feret diameter and particle size, reflecting elongated, vessel-like morphology. Together, these features effectively capture key differences in spatial and geometric patterns, supporting their utility for distinguishing analyzable vascular images from degraded ones.


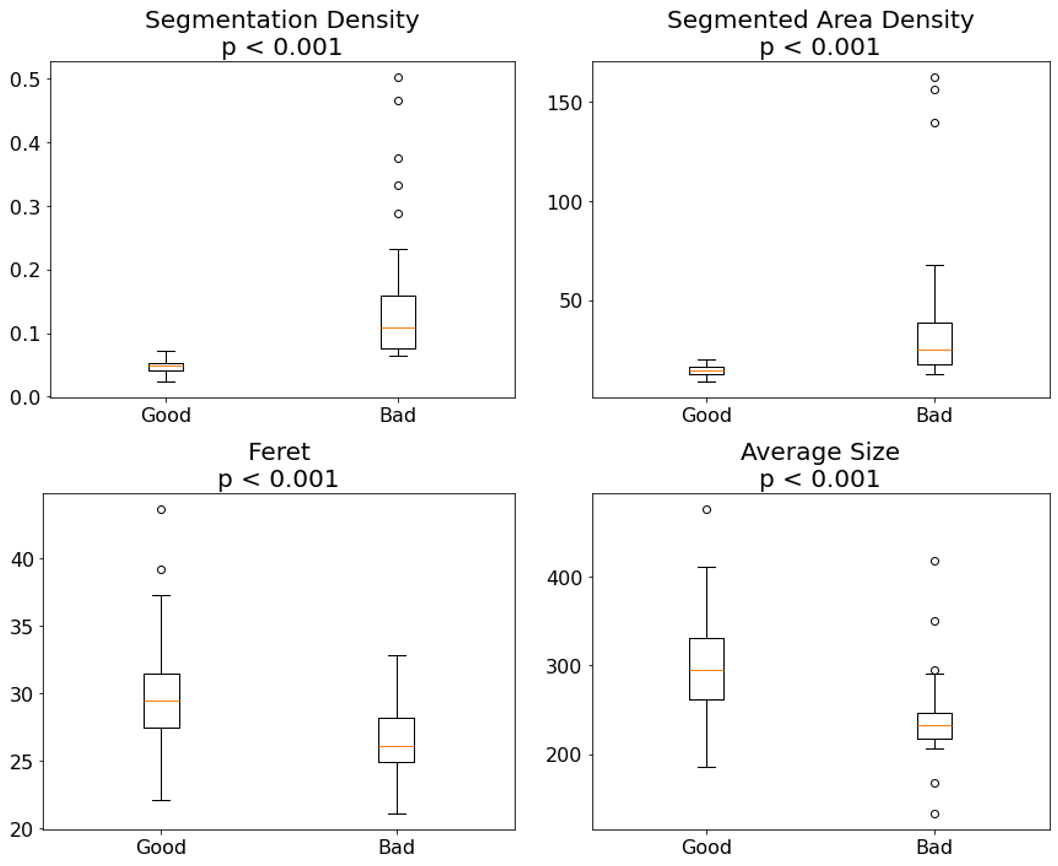


Figure S2. Quantitative comparison of morphological features between analyzable and non-analyzable OR-PAM images. Boxplots show four key features extracted from segmented binary masks: segmentation density, segmented area density, Feret diameter, and average particle size. All group differences were statistically significant with $p<0.001$ (Wilcoxon rank-sum test).

To objectively validate that our visual exclusions reflected quantifiable differences, we fitted a logistic regression classifier using the four selected features. These features were first standardized and projected into a two-dimensional principal component space with PCA. As shown in Figure S3 (a), analyzable and non-analyzable images exhibit clear clustering in PCA space, with the decision boundary effectively separating the two groups. The corresponding confusion matrix in Figure S3 (b) shows strong agreement with manual labels, correctly classifying 39 out of 40 analyzable images and 26 out of 28 non-analyzable images. These findings quantitatively confirm our visual assessments and support the use of this feature-based approach for automated or semi-automated image quality control. By providing objective criteria for identifying non-analyzable OR-PAM images, this pipeline can improve the robustness and reproducibility of future studies using vascular phenotyping for disease classification.

**
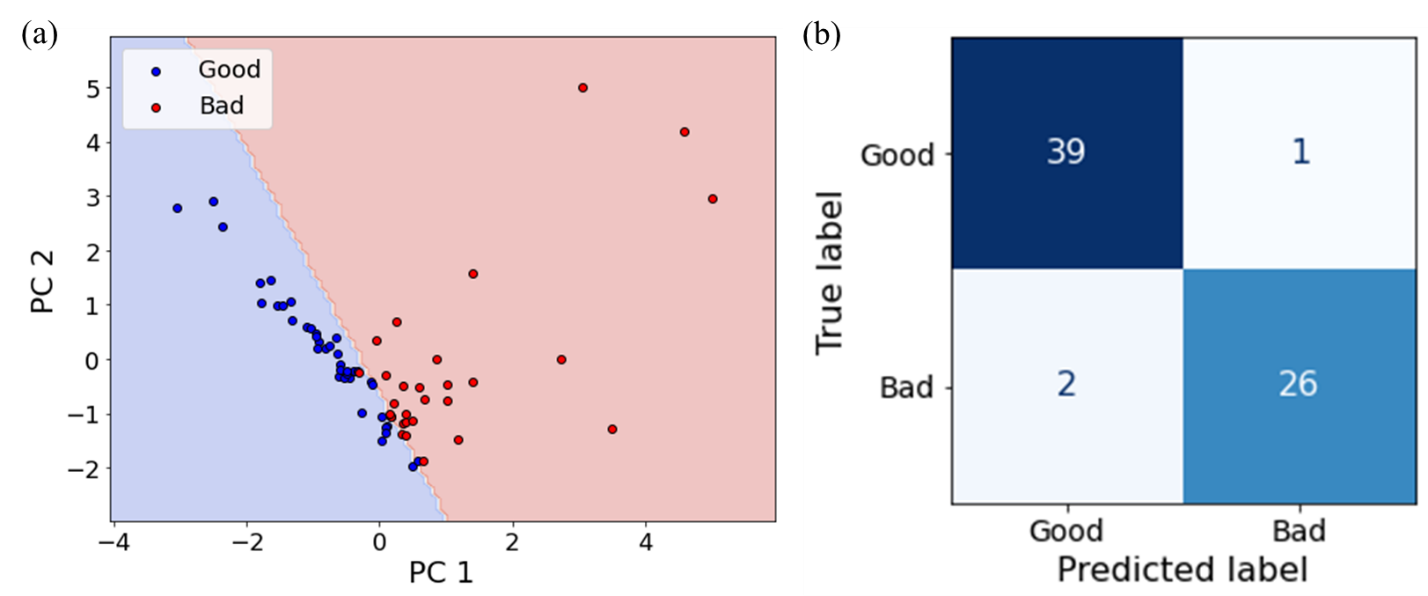
**

Figure S3. Performance of logistic regression classifier for image quality assessment based on extracted morphological features. (a) Decision boundary in principal component space after PCA dimensionality reduction of four features. Each point represents an OR-PAM image, colored by ground truth label. The shaded regions indicate classification zones predicted by the fitted model. (b) Confusion matrix summarizing classification performance. The model correctly identified 39 of 40 good scans and 26 of 28 bad scans, demonstrating strong overall classification accuracy.

**Supplementary Note 2: Quantitative Feature Extraction and Correlation Analysis**

A total of $31$ quantitative features were extracted from OR-PAM images to characterize vascular morphology, vascular network topology, and spectral properties of the photoacoustic signals. These features were derived from three complementary analysis pipelines: morphology analysis, vascular network analysis, and frequency-domain analysis. The complete list of extracted features and their descriptions is provided in Supplementary Table S1.

Supplementary Table S1. Summary of quantitative features extracted from OR-PAM images

| Feature Category | Feature Name | Description |
| --- | --- | --- |
| Morphology Analysis | Mean vessel diameter | Average vessel caliber |
|  | Vessel diameter SD | Variability of vessel diameter |
|  | Mode diameter | Most common vessel diameter |
|  | Median diameter | Median vessel diameter |
|  | Maximum diameter | Largest detected vessel |
|  | Diameter skewness | Asymmetry of size distribution |
|  | Diameter kurtosis | Spread of extreme vessel sizes |
|  | Total vessel length | Sum of vessel lengths in ROI |
|  | Integrated vessel area | Cumulative vessel area contribution |
|  | Raw integrated vessel area | Non-normalized vessel signal sum |
|  | Percent porosity | Fraction of non-vascular area |
| Vascular Network Analysis | Endpoint density (norm.) | Density of terminal vessel ends |
|  | Node density (norm.) | Density of all network nodes |
|  | Junction density (norm.) | Density of branching points |
|  | Master junction density (norm.) | Density of major junctions |
|  | Master segment density (norm.) | Density of principal vessel segments |
|  | Master segment length (norm.) | Length of main vessels per area |
|  | Mesh density (norm.) | Density of closed vessel loops |
|  | Mesh area density (norm.) | Area enclosed by loops per area |
|  | Segment density (norm.) | Density of all skeleton segments |
|  | Branch density (norm.) | Density of branch segments between junctions |
|  | Isolated segment density (norm.) | Density of disconnected vessels |
|  | Total vessel length (norm.) | Total skeleton length per area |
|  | Branching length (norm.) | Length of branching segments |
|  | Segment length (norm.) | Total skeleton segment length per area |
|  | Branch length (norm.) | Length of branch segments |
|  | Isolated branch length (norm.) | Length of disconnected branches |
|  | Piece density (norm.) | Density of disconnected vessel subnetworks |
|  | Branching interval | Mean distance between branch points |
|  | Mesh index | Measure of loop formation/connectivity |
| Frequency Analysis | Mean PA frequency | Amplitude-weighted spectral mean related to absorber size and microstructure |

*(norm.) denotes normalization by ROI image area

Morphology features quantify vessel size distribution and spatial organization using DiameterJ-based vessel segmentation. Network topology features describe connectivity and branching patterns of the vascular network obtained from skeletonized vessel maps using the Angiogenesis Analyzer plugin. Frequency features characterize spectral properties of the photoacoustic signals, which reflect effective absorber dimensions and tissue microstructure.

Following feature extraction, statistical screening was performed using Welch’s t-tests to identify features that significantly differed between normal/benign and EC/EIN samples. To reduce redundancy among candidate features, pairwise correlation analysis was subsequently performed. The five most statistically significant and minimally correlated features were selected for downstream analysis and separability assessment.

The pairwise correlation matrix of the five selected features is shown in Supplementary Figure S4. The matrix demonstrates only low to moderate correlations between features, confirming that the selected feature set captures complementary aspects of vascular morphology and spectral properties rather than redundant information.


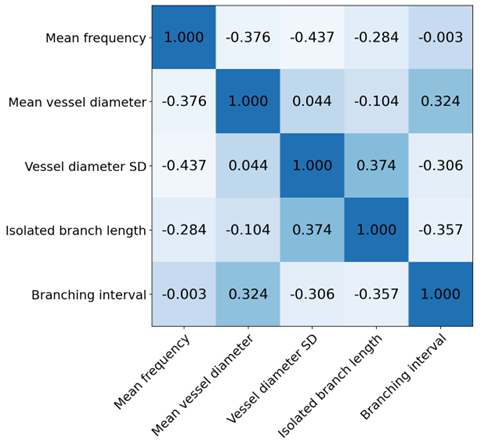


Figure S4. Pairwise correlation matrix of the selected features, showing low to moderate correlations and minimal redundancy among features.

**Supplementary Note 3: Dynamic Visualization of Feature-Space Organization and Class Separation**

To illustrate how OR-PAM images organize within the learned feature space used for classification, we provide a dynamic visualization that progressively reveals structural relationships between cases, as shown in Supplementary Video S1. Each image is represented by a node whose position is determined by pairwise cosine similarity of its five selected features. Similarity between cases is rendered as a weighted graph: edges connect pairs of specimens whose similarity exceeds a gradually decreasing threshold, beginning at 1.00 and ending at 0.50.

The video begins by showing all images as unlabeled grey nodes arranged in a unit-circle configuration. As the similarity threshold decreases, edges emerge between increasingly similar cases, revealing the intrinsic topology of inter-patient vascular feature similarity without using diagnostic labels. Once network structure is established, categorical labels (Normal/Benign vs. EC/EIN) are revealed, demonstrating that malignant and premalignant cases form a distinct cluster in this high-dimensional representation. Finally, the linear decision boundary from the logistic regression model is displayed, showing that the classifier’s separating hyperplane closely aligns with the naturally occurring separation suggested by similarity connectivity, indicating that the observed discrimination between groups arises from inherent feature-space structure rather than model artifacts.

Together, this visualization provides an intuitive interpretation of the embedding used for classification and offers insight into why relatively simple linear modeling is sufficient to achieve accurate diagnostic separation when using biologically informed vascular metrics.


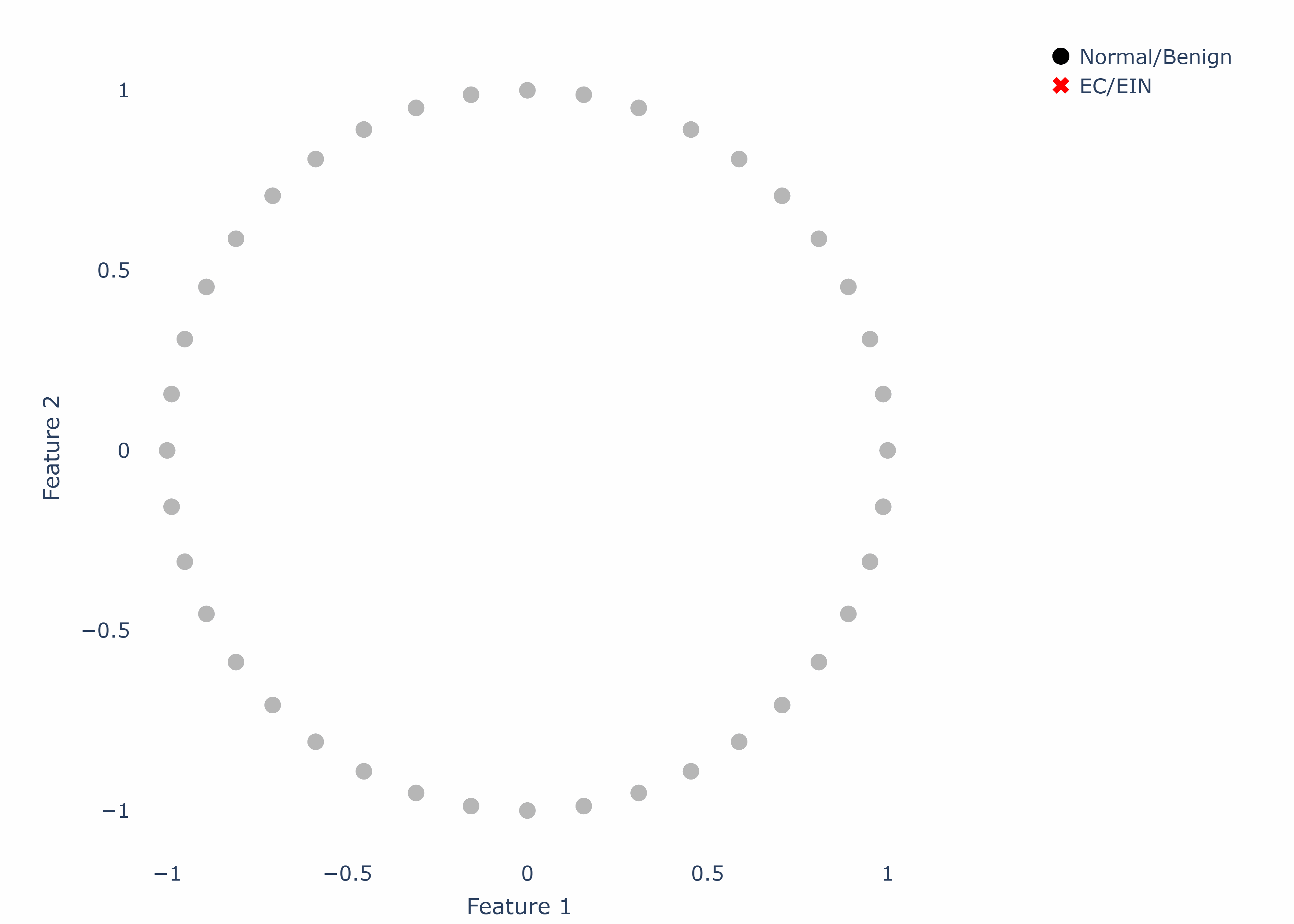


Supplementary Video S1. Animated visualization of the feature-based similarity embedding used for classification of endometrial pathology.

**Supplementary Note 4: Feature Analysis of the Misclassified Case**

Figure S5 provides a feature level visualization of the two normal/benign cases that were misclassified as EC/EIN by LOOCV. For several features, these cases exhibit values that overlap with or fall within the EC/EIN distribution rather than clustering with other normal/benign cases. In particular, both samples show increased vascular heterogeneity and network fragmentation compared with the majority of normal/benign cases. As a result, their combined feature profiles shift toward the EC/EIN region in the multidimensional feature space used for classification. These examples illustrate how the joint behavior of multiple vascular features can influence the model decision boundary and lead to overlap between groups. Accordingly, Figure S5 is intended to provide contextual insight into the multi-feature decision process of the classification framework rather than to serve as an independent diagnostic assessment.


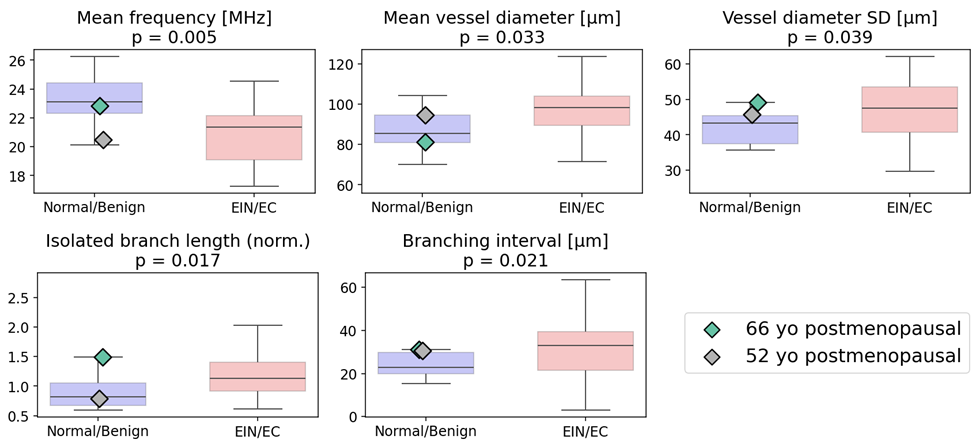


Figure S5. Boxplots of the five selected features with the two misclassified cases (a 66-year-old postmenopausal woman and a 52-year-old postmenopausal woman) highlighted.
